# Association of Driver Oncogene Variations With Outcomes in Patients With Locally Advanced Non–Small Cell Lung Cancer Treated With Chemoradiation and Consolidative Durvalumab

**DOI:** 10.1001/jamanetworkopen.2022.15589

**Published:** 2022-06-06

**Authors:** Yufei Liu, Zhe Zhang, Waree Rinsurongkawong, Carl M. Gay, Xiuning Le, Matthew S. Ning, Jeff Lewis, Vadeerat Rinsurongkawong, J. Jack Lee, Jack Roth, Stephen Swisher, Saumil Gandhi, Percy P. Lee, Don L. Gibbons, Ara A. Vaporciyan, John V. Heymach, Jianjun Zhang, Steven H. Lin

**Affiliations:** 1 Department of Radiation Oncology, The University of Texas MD Anderson Cancer Center, Houston; 2 Department of Sociology, Rice University, Houston, Texas; 3 Department of Biostatistics, The University of Texas MD Anderson Cancer Center, Houston; 4 Department of Thoracic/Head and Neck Medical Oncology, The University of Texas MD Anderson Cancer Center, Houston; 5 Department of Thoracic and Cardiothoracic Surgery, The University of Texas MD Anderson Cancer Center, Houston

## Abstract

**Importance:**

Consolidative durvalumab after definitive chemoradiation for unresectable locally advanced non–small cell lung cancer (NSCLC) can significantly improve progression-free survival (PFS) and overall survival (OS), as shown in the PACIFIC trial. However, whether patients with driver variations derive equal benefit from this regimen remains unclear.

**Objectives:**

To compare outcomes of patients with locally advanced NSCLC with and without driver variations treated with the PACIFIC regimen.

**Design, Setting, and Participants:**

This cohort study examined 104 patients with unresectable locally advanced NSCLC with mutational profiling treated at a tertiary cancer center with definitive chemoradiation and consolidative durvalumab from June 2017 through May 2020. Patients with recurrent disease or those receiving postoperative therapy were excluded. Outcomes were analyzed with Kaplan-Meier and multivariate regression analyses.

**Exposures:**

Patients were grouped according to the presence of non–*KRAS* driver variations (*EGFR* exon 19 deletion, *EGFR* exon 20 insertion, *EGFR* exon 21 mutation [L858R], *ERBB2* exon 20 insertion, *EML4-ALK* fusion, *MET* exon 14 skipping, *NTRK2* fusion), *KRAS* driver variations, or no driver variations.

**Main Outcomes and Measures:**

The primary outcomes were PFS, OS, and second progression-free survival (PFS2) times.

**Results:**

The 104 patients had a median (IQR) age of 65.1 (9.8) years, with 55 females (53%) and 85 former or current smokers (88%). There were 43 patients (41%) with driver variations with a median PFS time of 8.4 months vs 40.1 months for patients without driver variations (hazard ratio [HR], 2.75; 95% CI, 1.64-4.62; log-rank *P* < .001). Both patients with non–*KRAS* and *KRAS* driver variations had worse PFS. No difference in OS was found between patients with and without driver variations (log rank *P* = .24). Among the 63 patients who developed progressive disease, those with non–*KRAS* driver variations had a median PFS2 time of 13.7 months vs 4.4 months for all other patients (HR, 0.37; 95% CI, 0.21-0.64; log-rank *P* = .001). Rates of overall grade 2 toxic effects or higher did not differ by driver mutation status.

**Conclusions and Relevance:**

In this cohort study, driver variations in patients with unresectable locally advanced NSCLC were associated with significantly shorter PFS time after definitive chemoradiation and consolidative durvalumab. These findings suggest the need to consider additional or alternative treatment options to the PACIFIC regimen for patients with driver variations.

## Key Points

**Question:**

Do driver variations in unresectable locally advanced non–small cell lung cancer (NSCLC) alter clinical outcomes of treatment with definitive chemoradiation and consolidative durvalumab?

**Findings:**

In this cohort study of 104 patients with locally advanced non–small cell lung treated with definitive chemoradiation and consolidative durvalumab, *KRAS* and non–*KRAS* driver variations were associated with significantly shorter progression-free survival (8.4 months vs 40.1 months).

**Meaning:**

These findings suggest that chemoradiation and consolidative durvalumab is less beneficial for locally advanced NSCLC with driver variations; alternative therapies for these patients require consideration.

## Teaser

This cohort study compares outcomes of 104 patients with unresectable locally advanced non–small cell lung cancer with and without driver variations treated with the PACIFIC regimen.

## Introduction

The treatment of unresectable locally advanced non–small cell lung cancer (NSCLC), a disease with high recurrence rates and mortality, has been revolutionized by the phase 3 PACIFIC trial, which revealed that up to 1 year of consolidative therapy with durvalumab, an antiprogrammed cell death ligand 1 (PD-L1) agent, after chemoradiation (CRT) led to significantly improved progression-free survival (PFS) and overall survival (OS).^1,2^ However, the benefit of this regimen for patients with variations in driver oncogenes such as *EGFR* or *KRAS* is uncertain.^2,3^ Tyrosine kinase inhibitors (TKIs) have been developed against many non–*KRAS* variations and recently for *KRAS G12C* variations, resulting in promising outcomes.^4,5,6,7^

In this retrospective cohort analysis, we explored clinical outcomes of patients with unresectable locally advanced NSCLC who received CRT followed by consolidative durvalumab and stratified outcomes by the presence of non–*KRAS* driver variations, *KRAS* driver variations, or no driver variations. Additionally, we explored patterns of failure and salvage outcomes for patients who progressed and assessed treatment-related toxicities.

## Methods

### Study Design

The institutional review board at the University of Texas MD Anderson Cancer Center granted approval for this retrospective review, and all patients provided informed consent. Informed consent for treatments in the study are documented in the electronic medical record. Patients with unresectable locally advanced NSCLC who received concurrent CRT and at least 1 cycle of adjuvant durvalumab between June 2017 and May 2020 were identified by querying a single-institution database of patients with NSCLC with variation profiling. Of an initial 267 patients who fit these criteria, we excluded patients who were not treated definitively, did not receive durvalumab with consolidative intent, had stage IV disease, had recurrent disease, or received upfront surgery to yield a final total of 104 patients. Patients were grouped according to the presence or absence of driver variations (*EGFR* exon 19 deletion, *EGFR* exon 20 insertion, *ERBB2* exon 20 insertion, *EGFR* exon 21 variation [L858R], *ALK*-*EML4* fusion, *MET* exon 14 skipping, *NTRK2* fusion, or *KRAS* variation). The driver variation group was further subgrouped by *KRAS* or non–*KRAS* variations. Variation profiling was performed with next-generation sequencing panels of 50, 70, or 134 genes in our institutional molecular diagnostics laboratory.

Baseline demographic information (ie, age, sex, race, and smoking status), performance status, histology, staging, treatment details (ie, chemotherapy and radiation regimen), PD-L1 status, and clinical outcomes (ie, PFS, second progression-free survival [PFS2], and OS) were obtained from electronic medical records. Race was recorded based on the category of Asian, Black, Hispanic, Middle Eastern, or White as recorded in the electronic medical record. Race was considered on the basis that driver variations may have different prevalence based on race. Histologic classification of NSCLC was defined based on World Health Organization criteria.^8^ Disease staging was based on the eighth edition of the American Joint Committee on Cancer Staging Manual.^9^

### Study Outcomes

PFS was measured from the date of CRT completion to the date of disease recurrence, death from any cause, or last follow-up. We defined recurrence based on pathologic or radiographic standards as defined in the Response Evaluation Criteria in Solid Tumors (RECIST) version 1.1.^10^ OS was measured from the date of CRT completion to the date of death from any cause or last follow-up. For those with progressive disease, the PFS2 time was measured from the date of the first progression to the date of subsequent progression, death from any cause, or last follow-up. Patient follow-up in the medical record was recorded until December 1, 2021. Adverse effects were graded according to the Common Terminology Criteria for Adverse Effects (CTCAE) version 5.0.

### Statistical Analysis

PFS, PFS2, and OS across variation groups were evaluated with Kaplan-Meier survival curves, and hazard ratios (HRs) were generated using log-rank tests. Censoring was used for patients with incomplete follow-up data. Comparisons for PFS and OS were performed with 2 groups (ie, driver variations and no driver variations) and with 3 groups (ie, non–*KRAS* driver variations, *KRAS* driver variations, and no driver variations). Comparisons for PFS2 were performed with 2 groups (ie, non–*KRAS* drivers vs all other variations) and with 3 groups (ie, non–*KRAS* driver, *KRAS* drivers, and nondrivers). A subgroup analysis of PFS, OS, and PFS2 with patients without squamous cell histology was performed with Kaplan-Meier survival curves with HRs generated using log-rank tests.

Comparisons by patient characteristics were made with analysis of variance for continuous variables and with the Freeman-Fisher-Halton exact test for categorical variables. Control for confounding in PFS and PFS2 was performed using multivariate regression analysis with variation status, sex, smoking status, stage, age, performance status, PD-L1 status, and histology as independent variables. HRs for each variable were estimated from Cox proportional hazards regression models. Toxic effects rates, patterns of progression, and rates of oligoprogression (defined as 3 or fewer discrete lesions) were compared with the Freeman-Fisher-Halton exact test. Statistical significance was defined as a 2-sided *P* value of .05 or less. Kaplan-Meier curves were generated in Prism version 9 (GraphPad). Stata version 15 (StataCorp) was used for all statistical tests.

## Results

For the 104 patients in our cohort (Table 1), 43 (41%) had driver variations and these patients were more likely than patients with nondriver variations to have adenocarcinoma histology (37 [86%] vs 29 [48%]; *P* < .001), be female (31 [72%] vs 24 [39%]; *P* = .004), be an Asian, Black, Hispanic, or Middle Eastern individual (10 [23%] vs 6 [10%]; *P* = .013) and be never smokers (10 [23%] vs 2 [3%]; *P* < .001). Among patients with non–*KRAS* driver variations, *EGFR* variations were the most common (12 [57%]). Specific variations for all patients are listed in the eTable 1 in the Supplement. Of patients with driver variations, 33 (77%) had variation profiling performed prior to starting chemoradiation. The median (IQR) follow-up for the cohort was 23.6 (14.3-33.4) months.

**Table 1.** Patient Characteristics Stratified by Variation Status

Characteristic	No. (%)				*P* value
	All patients (n = 104)	Non–*KRAS* driver variations (n = 21)	*KRAS* driver variations (n = 22)	Nondriver variations (n = 61)	
Age at completion of CRT, mean (SD), y	65.1 (9.8)	63.8 (11.0)	66.8 (9.1)	65.0 (9.7)	.59
Sex					
Male	49 (47)	6 (29)	6 (27)	37 (61)	.004
Female	55 (53)	15 (71)	16 (73)	24 (39)	
Race					.013
Asian	4 (4)	4 (19)	0	0	
Black	8 (8)	1 (5)	3 (14)	4 (7)	
Hispanic	2 (2)	1 (5)	0	1 (2)	
Middle Eastern	2 (2)	0	1 (4)	1 (2)	
White	88 (85)	15 (71)	18 (82)	55 (90)	
Smoking status					<.001
Current	14 (13)	0	3 (14)	11 (18)	
Former	78 (75)	11 (52)	19 (86)	48 (79)	
Never	12 (12)	10 (48)	0	2 (3)	
Histology					<.001
Adenocarcinoma	66 (64)	17 (81)	20 (91)	29 (48)	
Squamous	33 (32)	3 (14)	0	30 (49)	
Adenosquamous	3 (3)	1 (5)	1 (5)	1 (2)	
Giant cell	1 (1)	0	1 (5)	0	
Sarcomatoid	1 (1)	0	0	1 (2)	
Disease stage at CRT					.62
IIB	6 (6)	0	1 (4)	5 (8)	
IIIA	37 (36)	8 (38)	8 (36)	19 (31)	
IIIB	53 (51)	9 (43)	12 (55)	32 (53)	
IIIC	10 (10)	4 (19)	1 (5)	5 (8)	
ECOG PS score at CRT					.38
0-1	95 (91)	21 (100)	20 (91)	54 (89)	
2	9 (9)	0	2 (9)	7 (11)	
CRT regimen					.11
Cisplatin/etoposide	2 (2)	1 (5)	1 (5)	0	
Cisplatin/pemetrexed	6 (6)	2 (10)	1 (5)	3 (5)	
Cisplatin/docetaxel	1 (1)	0	0	1 (2)	
Carboplatin/docetaxel	1 (1)	1 (5)	0	0	
Carboplatin/paclitaxel	88 (85)	14 (67)	20 (91)	54 (89)	
Carboplatin/pemetrexed	4 (4)	3 (14)	0	1 (2)	
Carboplatin/etoposide	1 (1)	0	0	1 (2)	
Carboplatin only	1 (1)	0	0	1 (2)	
Radiation dose, Gy					.85
60	12 (12)	3 (14)	2 (9)	7 (12)	
66	76 (73)	15 (71)	16 (73)	45 (74)	
68	1 (1)	0	1 (5)	0	
69.6	1 (1)	0	0	1 (2)	
70	2 (2)	0	0	2 (3)	
72	7 (7)	2 (10)	1 (4)	4 (7)	
77	1 (1)	0	1 (4)	0	
Unknown	4 (4)	1 (5)	1 (4)	2 (3)	
PD-L1 expression					.32
Negative (0% TPS)	11 (11)	4 (19)	4 (18.2)	3 (5)	
Low (1%-49% TPS)	36 (35)	6 (29)	6 (27.3)	24 (39)	
High (≥50% TPS)	23 (22)	3 (14)	6 (27.3)	14 (23)	
Not tested	34 (33)	8 (38)	6 (27.3)	20 (33)	
Follow-up, median, mo	23.6	32.6	21.9	23.8	.26

Abbreviations: CRT, chemoradiation therapy; ECOG PS, Eastern Cooperative Oncology Group performance status; PD-LI, antiprogrammed cell death ligand 1; TPS, tumor proportion score.

### PFS and OS After CRT With Consolidative Durvalumab

**Figure 1.**
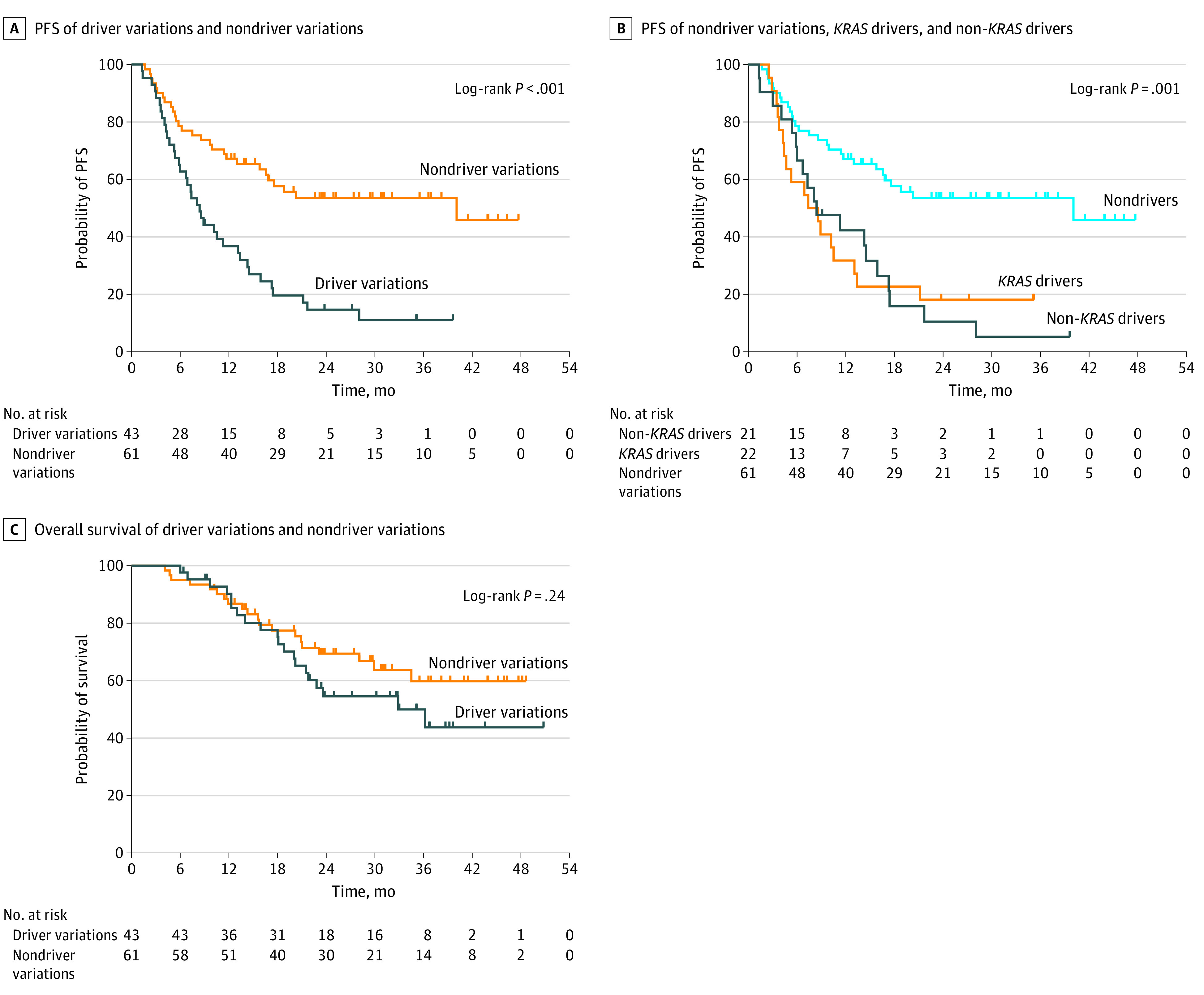
Progression-Free Survival (PFS) and Overall Survival for Patients With NSCLC With or Without Driver Variations Treated With Definitive Chemoradiation and Consolidative Durvalumab A, Median PFS time was 8.4 months for patients with driver variations vs 40.1 months in patients without driver variations (log-rank *P* < .001). B, Median PFS time was 8.4 months for the non–*KRAS* driver mutation group, 8.0 months for the *KRAS* driver mutation group, and 40.1 months for the non-driver mutation group (log-rank *P* = .001). C, Median OS time was 36.2 months for those with driver variations and was not achieved in those without driver variations (log-rank *P* = .24).

The median (IQR) PFS time for all patients was 14.5 months (5.7 months to not achieved). Patients with driver variations, both non–*KRAS* (8.4 months) and *KRAS* (8.0 months), had significantly shorter median PFS times (8.4 months vs 40.1 months; HR, 2.75; 95% CI, 1.64-4.62; *P* < .001) (Figure 1). On multivariate analysis, non–*KRAS* driver variation, *KRAS* driver variation, stage IIIC disease, and ECOG 2 were associated with worse PFS (Table 2). No significant difference was found in OS time between patients with driver variations (median [IQR], 36.2 months [IQR, 18.1 months to not achieved]) and those without (median not achieved) (*P* = .24) (Figure 1).

**Table 2.** Multivariate Regression Analysis for Progression-Free Survival

Characteristic	Hazard ratio (95% CI)	*P* value
Mutation status		
Non-driver	1 [Reference]	NA
Non-*KRAS* driver	5.12 (2.21-11.86)	<.001
*KRAS* driver	5.79 (2.69-12.47)	<.001
Sex		
Male	1 [Reference]	NA
Female	0.6 (0.33-1.09)	.09
Smoking status		
Former or current	1 [Reference]	NA
Never	0.93 (0.37-2.37)	.89
Stage		
IIB	1 [Reference]	NA
IIIA	0.92 (0.20-4.26)	.92
IIIB	2.97 (0.69-12.86)	.15
IIIC	7.88 (1.29-48.27)	.03
Age		
<65	1 [Reference]	NA
≥65	0.97 (0.54-1.75)	.93
ECOG		
0-1	1 [Reference]	NA
2	3.4 (1.35-8.53)	.009
PD-L1		
Negative (0% TPS)	1 [Reference]	NA
Low (1%-49% TPS)	1.02 (0.42-2.48)	.96
High (≥50% TPS)	0.62 (0.22-1.71)	.35
Not tested	0.67 (0.26-1.75)	.42
Histology		
Adenocarcinoma	1 [Reference]	NA
Nonadenocarcinoma	1.72 (0.91-3.27)	.10

Abbreviations: ECOG, Eastern Cooperative Oncology Group; PD-LI, antiprogrammed cell death ligand 1; TPS, tumor proportion score.

Because squamous histology may bias outcomes and variation testing on patients with NSCLC and squamous histology is not routinely performed at all institutions, we performed a subgroup analysis for all patients without nonsquamous histology. The subgroup analysis showed that driver variations still were associated with significantly worse PFS (median 8.5 months vs not achieved; HR, 4.49; 95% CI, 2.47-8.19; *P* < .001) (eFigure 1 in the Supplement). There continued to be no significant difference in OS (median 36.2 months for drivers vs not achieved in nondrivers; HR, 2.16; 95% CI, 0.95-4.9; 1 *P* = .07) (eFigure 1 in the Supplement).

Patterns of failure were not significantly different between patients with and without driver variations, with distant failures accounting for the majority of first progressions (23 [64%] vs 16 [59%]; *P* = .71) in both groups. Rates of oligometastatic progression (defined as 3 or fewer sites of progression) were also not significantly different (driver variations vs nondriver variations, 10 [28%] vs 13 [48%]; *P* = .12).

### Time to Second Progression

**Figure 2.**
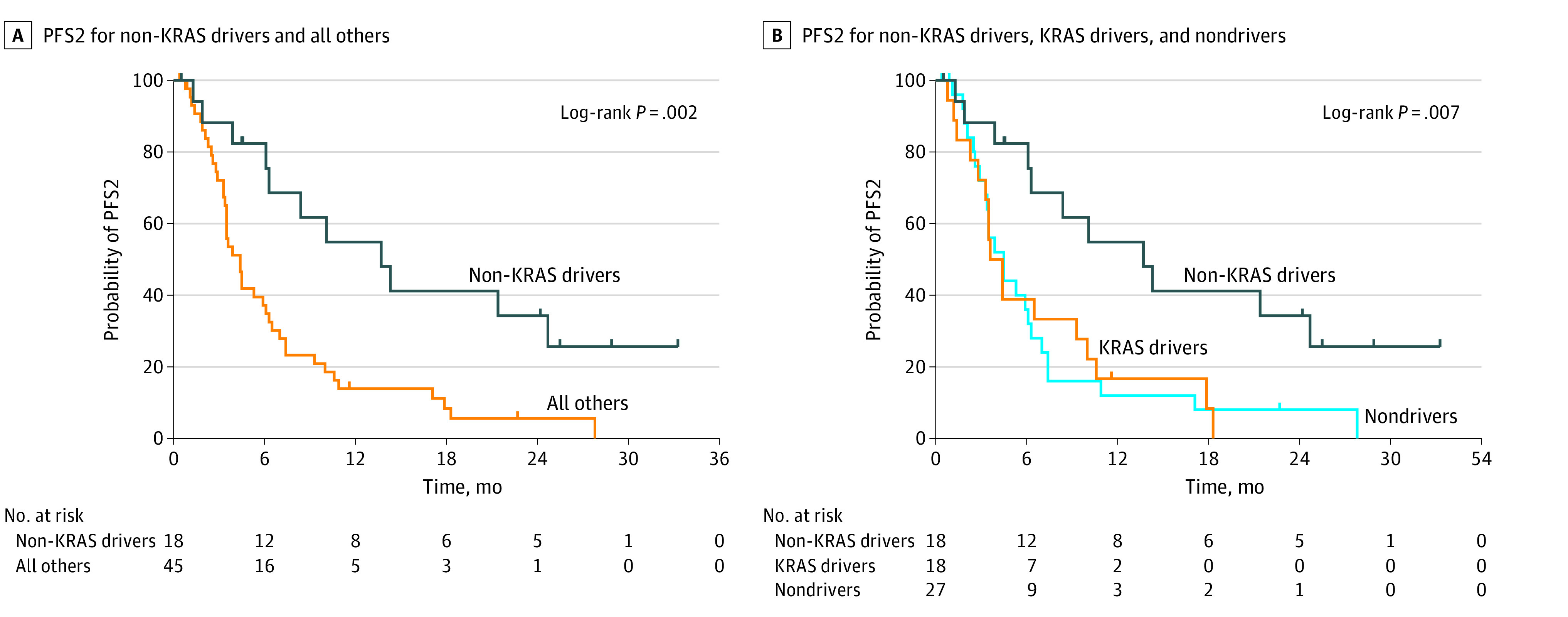
Time to Second Progression (PFS2) in Patients With or Without Driver Variations Who Progressed After Definitive Chemoradiation and Consolidative Durvalumab A, Median time after the first episode of disease progression (PFS2) was 13.7 months for patients with non–*KRAS* driver variations vs 4.4 months for all other patients (log-rank *P* = .002). B, Median PFS2 time was 13.7 months for patients with non–*KRAS* driver variations, 4.0 months for patients with *KRAS* driver variations, and 4.5 months for patients without driver variations (log-rank *P* = .007).

We next addressed PFS2 for patients that progressed post-CRT and at least 1 cycle of durvalumab. Patients with non–*KRAS* driver variations had significantly longer PFS2 (median [IQR], 13.7 months [6.3 months to not achieved]) relative to all other patients (median [IQR], 4.4 [2.9-7.4] months) (HR, 0.37; 95% CI, 0.21-0.64; *P* = .002) (Figure 2). For the multivariate analysis, non–*KRAS* driver variation was the only factor associated with improved PFS2 (eTable 2 in the Supplement). For the subgroup analysis of patients without squamous histology, non–*KRAS* driver variations was still associated with significantly longer PFS2 (median [IQR], 14.3 months [8.4 months to not achieved]) compared with all other patients (median [IQR], 3.9 [2.6-10.0] months) (HR, 0.33; 95% CI, 0.17-0.64; *P* = .002) (eFigure 2 in the Supplement). All patients with non–*KRAS* driver variations and 1 patient with *KRAS G12C* variation received TKI therapy as part of their postrelapse therapy with many receiving it as their first line of treatment (eTable 1 in the Supplement).

### Treatment-Related Toxic Effects

Rates and grades of toxic side effects are summarized in Table 3. The overall rate of grade 2 or higher toxic effects among all patients was 75%, with 23% experiencing grade 3 or higher toxic effects. The rates of both grade 2 or higher toxic effects (*P* = .78) and grade 3 or higher toxic effects (*P* = .77) did not differ by driver variation status.

**Table 3.** Treatment Toxic Effects by Variation Status

Toxic Effects	No. (%)				*P* value
	All patients (n = 104)	Non-*KRAS* driver variations (n = 21)	*KRAS* driver variations (n = 22)	Nondriver variations (n = 61)	
**All toxicities**					
Grade 2 or higher	78 (75.0)	17 (81.0)	17 (77.3)	44 (72.1)	.78
Grade 3 or higher	24 (23.1)	6 (28.6)	5 (22.7)	13 (21.3)	.77
**Pneumonitis**					
Grade 2 or higher	44 (42.3)	13 (61.9)	10 (45.5)	21 (34.4)	.09
Grade 3 or higher	17 (16.3)	4 (19.0)	3 (13.6)	10 (16.4)	.87
**Dysphagia**					
Grade 2 or higher	30 (28.8)	4 (19.0)	6 (27.3)	20 (32.8)	.53
Grade 3 or higher	0	0	0	0	> .99
**Esophagitis**					
Grade 2 or higher	48 (46.2)	9 (42.9)	9 (40.9)	30 (49.2)	.80
Grade 3 or higher	2 (1.9)	0	0	2 (3.3)	> .99
**Pain**					
Grade 2 or higher	25 (24.0)	4 (19.0)	3 (13.6)	18 (29.5)	.30
Grade 3 or higher	3 (2.9)	0	0	3 (4.9)	.57
**Dermatitis**					
Grade 2 or higher	12 (11.5)	2 (9.5)	2 (9.1)	8 (13.1)	> .99
Grade 3 or higher	2 (1.9)	1 (4.8)	0	1 (1.6)	.41
**Arthritis**					
Grade 2 or higher	1 (1.0)	1 (4.8)	0	0	.202
Grade 3 or higher	0	0	0	0	> .99
**Diarrhea**					
Grade 2 or higher	2 (1.9)	1 (4.8)	1 (4.5)	0	.169
Grade 3 or higher	2 (1.9)	1 (4.8)	1 (4.5)	0	.17
**Anorexia**					
Grade 2 or higher	6 (5.8)	0	1 (4.5)	5 (8.2)	.62
Grade 3 or higher	1 (1.0)	0	0	1 (1.6)	> .99
**Dehydration**					
Grade 2 or higher	3 (2.9)	0	0	3 (4.9)	.57
Grade 3 or higher	1 (1.0)	0	0	1 (1.6)	> .99
**Fatigue**					
Grade 2 or higher	9 (8.7)	0	2 (9.1)	7 (11.5)	.38
Grade 3 or higher	0	0	0	0	> .99

## Discussion

In this cohort study of patients with unresectable locally advanced NSCLC with driver variations, we found that the presence of a driver variation was associated with significantly worse PFS with the standard of care regimen of definitive chemoradiation and consolidative durvalumab, which is consistent with previous studies.^1,2,3^ We also found that for patients with targetable driver variations who progress, TKIs are an effective salvage regimen.

Our results lead to the question of why patients with locally advanced NSCLC with driver variations have such poor outcomes with the PACIFIC regimen. A possible explanation is that oncogene-driven NSCLC may have a smaller tumor mutation burden (TMB) compared with nondriver NSCLC given their addiction to particular signaling pathways. While an extensive analysis of TMB has not been performed across a large set of driver variations in NSCLC, a study has shown that *EGFR*-variant NSCLC has markedly lower TMB compared with *EGFR*-wildtype NSCLC.^11^ Lower TMB has been shown in multiple studies to predict worse outcomes on immune checkpoint inhibitors, including in the CheckMate 227 trial.^12,13^ This is possibly because of fewer immunogenic targets. More work remains to be done to explore the relationship between driver variations and TMB in NSCLC, which could potentially guide the future use of immunotherapy.

Several studies have assessed whether TKIs can improve clinical outcomes in patients with NSCLC with driver variations. A previous retrospective study^3^ reported significantly improved PFS among patients with *EGFR*-mutated stage III NSCLC who were given induction or consolidative *EGFR* TKI in conjunction with CRT compared with the PACIFIC regimen (26.1 months vs 10.3 months). The phase 3 ADAURA trial showed significantly improved disease-free survival with adjuvant osimertinib (89% vs 52%) in resected stage IB to stage IIIA *EGFR*-mutated NSCLC.^14^ The phase 3 LAURA trial is currently investigating consolidative osimertinib for patients with unresectable stage III NSCLC after CRT.^15^ However, use of TKIs in combination with immunotherapy must be pursued carefully given reports of increased toxic effects.^16,17,18^

### Limitations

This study has limitations. A major limitation of our study was the small sample size and retrospective format from a single institution, which could carry some inherent biases to the outcomes and conclusion. Additionally, given the relatively recent adoption of consolidative durvalumab as the standard of care, our cohort has relatively short follow-up time and immature OS data. Despite these limitations, our study is one of the largest assessments of the effectiveness of the PACIFIC regimen in unresectable locally advanced NSCLC patients with driver variations.

## Conclusions

The findings of this cohort study highlight the prognostic importance of assessing gene variation status in unresectable locally advanced NSCLC patients in guiding treatment decisions. Our data suggest the need for future clinical trials to test the potential benefit of replacing or combining durvalumab with TKI therapy for patients with driver variations.

## Notes

Supplement. **eTable 1. **Variations and Salvage Therapies of All Patients **eTable 2. **Multivariate Regression Analysis for Time to Second Progression **eFigure 1. **Progression-Free Survival (PFS) and Overall Survival (OS) for Nonsquamous NSCLC Patients With or Without Driver Variations Treated With Definitive Chemoradiation and Consolidative Durvalumab **eFigure 2. **Time to Second Progression (PFS2) in Non-Squamous Patients With or Without Driver Variations Who Progressed After Definitive Chemoradiation and Consolidative Durvalumab
