# Supplementary material for: Association of Driver Oncogene Variations With Outcomes in Patients With Locally Advanced Non–Small Cell Lung Cancer Treated With Chemoradiation and Consolidative Durvalumab
Source: JAMA Netw Open. 2022 Jun 6;5(6):e2215589. doi: 10.1001/jamanetworkopen.2022.15589 (PMC9171557; doi:10.1001/jamanetworkopen.2022.15589)
Supplement: Supplement. — eTable 1. Variations and Salvage Therapies of All Patients eTable 2. Multivariate Regression Analysis for Time to Second Progression eFigure 1. Progression-Free Survival (PFS) and Overall Survival (OS) for Nonsquamous NSCLC Patients With or Without Driver Variations Treated With Definitive Chemoradiation and Consolidative Durvalumab eFigure 2. Time to Second Progression (PFS2) in Non-Squamous Patients With or Without Driver Variations Who Progressed After Definitive Chemoradiation and Consolidative Durvalumab [file jamanetwopen-e2215589-s001.pdf]

Supplemental Online Content

Liu Y, Zhang Z, Rinsurongkawong W, et al. Association of driver oncogene variations with outcomes in patients with locally advanced non–small cell lung cancer treated with chemoradiation and consolidative durvalumab. *JAMA Netw Open*. 2022;5(6):e2215589. doi:10.1001/jamanetworkopen.2022.15589

eTable 1. Variations and Salvage Therapies of All Patients
eTable 2. Multivariate Regression Analysis for Time to Second Progression
eFigure 1. Progression-Free Survival (PFS) and Overall Survival (OS) for Nonsquamous NSCLC Patients With or Without Driver Variations Treated With Definitive Chemoradiation and Consolidative Durvalumab
eFigure 2. Time to Second Progression (PFS2) in Non-Squamous Patients With or Without Driver Variations Who Progressed After Definitive Chemoradiation and Consolidative Durvalumab

This supplemental material has been provided by the authors to give readers additional information about their work.

© 2022 Liu Y et al. *JAMA Network Open.*

eTable 1. Variations and Salvage Therapies of All Patients

UID	Driver Mutation	Other Identified Mutations	Progression?	TKI Therapy (* - first post-relapse therapy)	Other Salvage Therapy (* - first post-relapse therapy)
7008	EML4-ALK Fusion	None	Y	Alectinib*	
7440	EML4-ALK Fusion	None	Y	Alectinib*	
8105	EML4-ALK Fusion	None	N	No progression	
6504	EML4-ALK Fusion	MTOR	Y	Brigatinib*	
7865	EML4-ALK Fusion	TP53, MLH1, BRCA2	Y	Alectinib*	
6075	EML4-ALK Fusion	TP53	Y	Alectinib*	
8017	EGFR exon 19 deletion	None	Y	Osimertinib*	
6035	EGFR exon 19 deletion	TP53, ARID1A, CCNE1, MYC, PIK3CA, TSC1, ATM	Y	Osimertinib*	
7333	EGFR exon 19 deletion	TP53, PIK3CA, RB1	Y	Osimertinib	Radiation*
5755	EGFR exon 19 deletion	TP53	Y	Osimertinib*	
8200	EGFR exon 19 deletion	TP53, NOTCH1	Y	Osimertinib	Radiation*
5779	EGFR exon 19 deletion	CTNNB1, MET	Y	Osimertinib*	
4914	EGFR exon 19 deletion	KDR, PIK3CA	N		
7452	EGFR exon 20 insertion	TP53, cMET amp, RB1	Y	Poziotinib	Chemoradiation*
5516	EGFR exon 21, L858R	TP53, CDK6	Y	Osimertinib	
5585	EGFR exon 21, L858R	TP53	Y	Osimertinib (Induction), Neratininb/Palbociclib	Carboplatin/Pemetrexed/Bevacizumab*
5932	EGFR exon 21, L858R	TP53, PIK3CA, PTEN, RB1	Y	Osimertinib*	Radiation/Surgery
6000	EGFR exon 21, L858R	JAK2, NF1	Y	Osimertinib (Induction and salvage)	Radiation/Surgery*
7785	HER2 exon 20 insertion	MAP2K4	Y	Poziotinib*	
7030	MET exon 14 skipping	None	N		
7217	NTRK2 fusion	TP53, ARID1A, EGFR exon 3, FANCA, FGFR4, MSH6, NFE2L2, PIK3CA, RNF43, SMARCB1	Y	Larotrectinib	Radiation*
7497	KRAS G12A	TP53, CREBBP, NF2	Y		Radiation/Surgery*
6682	KRAS G12C	None	N		
6564	KRAS G12C	STK11, CCNE1	Y		Radiation*
5353	KRAS G12C	ATM, NOTCH1, PALB2, TERT	Y		Radiation*
8610	KRAS G12C	STK11	Y		Taxotere/Ramucirumab*
6126	KRAS G12C	TP53, STK11, APC, CDH1, CDKN2A, FGFR1, MYC, TET2	Y		Radiation/AZD9150*

© 2022 Liu Y et al. *JAMA Network Open.*

UID	Driver Mutation	Other Identified Mutations	Progression?	TKI Therapy (* - first post-relapse therapy)	Other Salvage Therapy (* - first post-relapse therapy)
7956	KRAS G12C	TP53, ARID1A, BRCA1	Y		Carboplatin/Pemetrexed/Pembrolizumab*
7376	KRAS G12C	ARID1A	Y		Radiation*
4332	KRAS G12C	ATM	N		
7439	KRAS G12C	TP53, STK11	Y	AMG510 (sotorasib)	Carboplatin/Pemetrexed/Pembrolizumab*
7629	KRAS G12D	TP53, ATR, BRCA2, PIK2CA	N		
6038	KRAS G12D	TP53, STK11, POLE	N		
7143	KRAS G12D	TP53	Y		Carboplatin/Pemetrexed/Pembrolizumab*
7891	KRAS G12D	STK11, NFE2L2	Y		Docetaxel/Ramucirumab*
6734	KRAS G12R	AKT3, IGF1R, NOTCH3, CTNNB1, U2AF1	Y		Radiation*, Carboplatin/Pemetrexed/Pembrolizumab
7601	KRAS G12V	STK11, KEAP1	Y		Carboplatin/Pemetrexed/Bevacizumab*
6939	KRAS G12V	STK11	Y		2018-0985 IPN60090*
8043	KRAS G12V	TP53, ATM, AXL, CREBBP, NOTCH2	Y		Carboplatin/Abraxane/Pembrolizumab*
6459	KRAS G13C	TP53	Y		Carboplatin/Paclitaxel*
8621	KRAS Q22K	STK11, ATM, ATRX	Y		Radiation*, PD1-TIM-3
6594	KRAS Q61H	TP53, ARID1A, U2AF1, FGFR4	Y		Ipilimumab/Nivolumab*
7955	KRAS Q61H	PTCH1	Y		Radiation*
6132	None	TP53, ATM, CDKN2A, FBXW1, PIK3R1	Y		Carboplatin/Paclitaxel*
8649	None	None	Y		Taxotere*
7414	None	TP53, ATR, CDKN2A, NFE2L2, PIK3CA, TERT	Y		Carboplatin/Paclitaxel/Pembrolizumab*
6767	None	TP53, FGFR1	Y		Carboplatin/Paclitaxel/Pembrolizumab*
8532	None	None	Y		JTX-2011/Ipilimumab*
4707	None	TP53, STK11, BRCA2, FGFR1	Y		Radiation/Surgery*
8261	None	None	Y		Pemetrexed/Pembrolizumab*
7384	None	None	Y		Surgery*
6577	None	TP53, EGFR amplification, FGFR2, SETD2, SMARCA4	Y		
8086	None	TP53, BRCA1	Y		Carboplatin/Pemetrexed*
5760	None	TP53, STK11, PTEN	Y		
7459	None	TP53, MAP2K1	Y		Carboplatin/Pemetrexed/Pembrolizumab*
7078	None	TP53	Y		
5788	None	TP53, ATM, CDKN2A, FLT3, ROS1, TERT	Y		Surgery*
7356	None	TP53, CDKN2A	Y		Carboplatin/Pemetrexed/Pembrolizumab*
5571	None	KDR, KIT, PIK3CA	Y		Radiation*
4483	None	TP53, APC, BRCA2, CCND1, PIK3R1	Y		NKTR-214/Nivolumab*
5096	None	ARID1A, BRCA1, FGFR3, MAP2K1, MTOR, PIK3CA	Y		Carboplatin/Pemetrexed/Pembrolizumab*
6893	None	None	Y		Carboplatin/Paclitaxel/Pembrolizumab*
7372	None	TP53, ATRX, MDM2, NOTCH2, RAD50, RAD51B, TSC2	Y		Chemoradiation*
5430	None	TP53	Y		Carboplatin/Abraxane/Atezolizumab*

© 2022 Liu Y et al. *JAMA Network Open.*

UID	Driver Mutation	Other Identified Mutations	Progression?	TKI Therapy (* - first post-relapse therapy)	Other Salvage Therapy (* - first post-relapse therapy)
4791	None	None	Y		
5122	None	None	Y		Radiation*
6193	None	TP53, AKT2, NFE2L2, NOTCH2, PIK3CA, SLX4	Y		Nivolumab*
7582	None	TP53, CDKN2A, FGFR3, NOTCH1	Y		Carboplatin/Pemetrexed/Pembrolizumab*
7599	None	STK11, CTNNB1	Y		Radiation*
5749	None	TP53, ATM, CDKN2A, EGFR exon 28	Y		Carboplatin/Paclitaxel*
8591	None	TP53, STK11, MYCL, TERT, CTNNB1, NBN, SMARC4	N		
5844	None	None	N		
8358	None	TP53, NTRK3, PPARG	N		
7450	None	TP53, STK11, AKT1, ARID1A, ATM, ATR, BAP1, BRAF E695Q, BRCA1, BRCA2, CCND2, CDK2, CDKN1B, CDKN2A, CREBBP, CSF1R, ERBB2, ERBB3, FANCA, FANCD2, FGFR4, KIT, MDM2, MRE11A, MSH2, MSH6, NF1, NOTCH2, NTRK1, PALB2, PDGFRB, PD-L1, PIK3CA, POLE, PTCH1, RAD50, RAD51D, RB1, SETD2, SMARCB1, TERT, TSC1, TSC2	N		
5025	None	APC, FGFR1	N		
6507	None	None	N		
7097	None	STK11	N		
7109	None	TP53, CDK6, KRAS amplification, RAF1	N		
8248	None	None	N		
6906	None	None	N		
4985	None	None	N		
8277	None	TP53, ATM, FLT3, PIK3CA	N		
5896	None	None	N		
7381	None	TP53, ARID1A, EGFR amplification, RICTOR, SMARCA1, TERT	N		
5569	None	TP53, KDR, PIK3CA	N		
5965	None	None	N		
7246	None	None	N		
6736	None	None	N		
5155	None	FBXW7, NOTCH1	N		
7808	None	None	N		

© 2022 Liu Y et al. *JAMA Network Open.*

UID	Driver Mutation	Other Identified Mutations	Progression?	TKI Therapy (* - first post-relapse therapy)	Other Salvage Therapy (* - first post-relapse therapy)
6641	None	None	N		
5491	None	TP53	N		
7128	None	None	N		
6666	None	None	N		
7815	None	BCORL1, KDM6A, PIGA, WT1	N		
7167	None	None	N		
7854	None	None	N		
7765	None	TP53, PTEN	N		
7539	None	None	N		
8196	None	TP53, AXL, BRCA2, DDR2, FANC1, KIT, NF1, PIK3CA, PIK3R1, SMO, TERT	N		
7665	None	None	N		
8692	None	None	N		
6726	None	TP53, BRCA2, CCND1, CDKN2A, FGF19, FGF13, FGFR1, KRAS amplification, NOTCH1, SETD2	N		
5072	None	TP53	N		

© 2022 Liu Y et al. *JAMA Network Open.*

eTable 2. Multivariate Regression Analysis for Time to Second Progression

Characteristic	Hazard Ratio	95% Confidence Interval	P-value
Mutation Status			
Non-Driver	1.00		
Non-KRAS Driver	0.29	0.11-0.78	0.01
KRAS Driver	1.07	0.42-2.74	0.88
Sex			
Male	1.00		
Female	0.53	0.25-1.10	0.09
Smoking Status			
Former or Current	1.00		
Never	0.93	0.31-2.79	0.90
Stage			
IIB	1.00		
IIIA	4.75	0.48-46.79	0.18
IIIB	3.29	0.36-29.94	0.29
IIIC	6.48	0.56-75.48	0.14
Age			
<65	1.00		
≥65	1.19	0.59-2.42	0.63
ECOG			
0-1	1.00		
2	1.06	0.40-2.84	0.91
PD-L1			
Negative (0% TPS)	1.00		
Low (1-49% TPS)	0.45	0.14-1.41	0.17
High (≥50% TPS)	0.82	0.24-2.83	0.75
Not Tested	1.02	0.33-3.13	0.98
Histology			
Adenocarcinoma	1.00		
Non-Adenocarcinoma	1.00	0.41-2.43	1.00

© 2022 Liu Y et al. *JAMA Network Open.*

eFigure 1. Progression-Free Survival (PFS) and Overall Survival (OS) for NSCLC Patients With or Without Driver Variations Treated With Definitive Chemoradiation and Consolidative Durvalumab

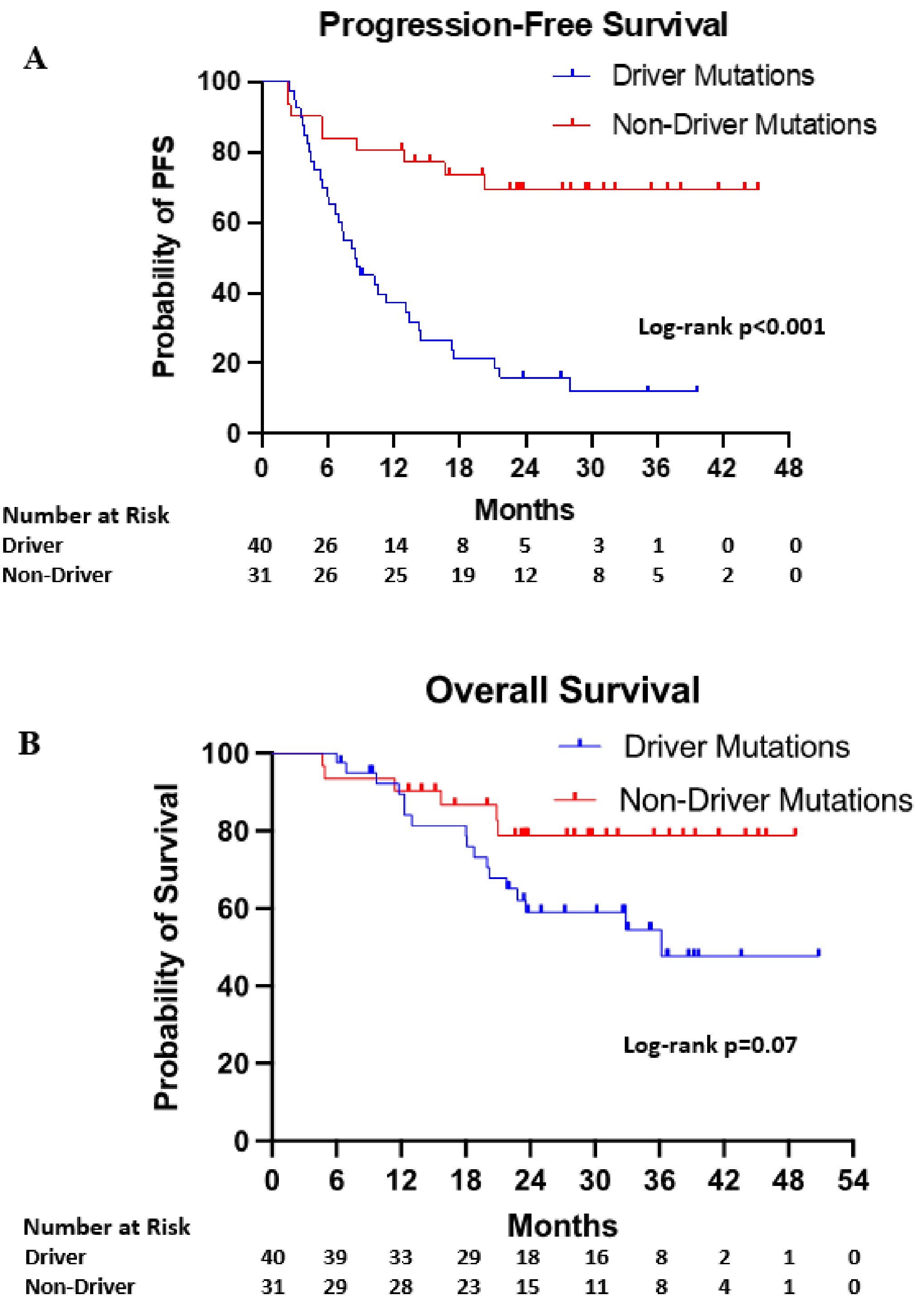

(A) Median PFS time was 8.5 months for patients with driver mutations versus not reached in patients without driver mutations (log-rank *P*<0.001). (B) Median OS time was 36.2 months for those with driver mutations and was not reached in those without driver mutations (log-rank *P*=0.07).

© 2022 Liu Y et al. *JAMA Network Open.*

eFigure 2. Time to Second Progression in Patients With or Without Driver Variations Who Progressed After Definitive Chemoradiation and Consolidative Durvalumab

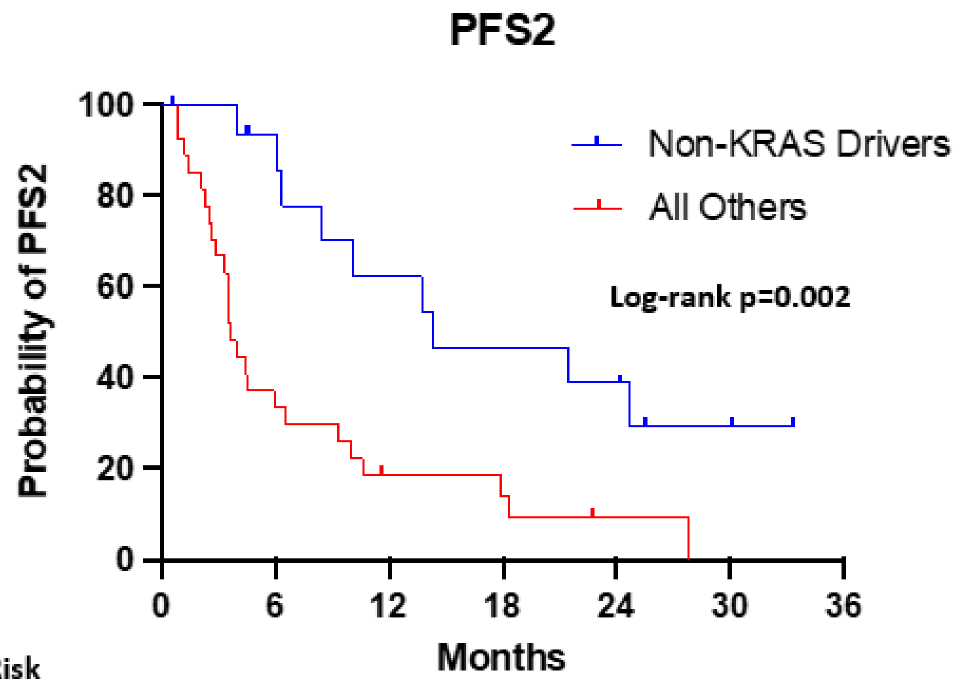

Number at Risk							
Non-KRAS Drivers	16	12	8	6	5	1	0
All Others	27	9	4	3	1	0	0

Median time to second disease progression (PFS2) was 14.3 months for patients with non-KRAS driver mutations versus 3.9 months for all other patients (log-rank *P*=0.002).

© 2022 Liu Y et al. *JAMA Network Open.*
